# Humanin and MOTS-c Attenuate Atrial Fibrillation by Suppressing Fibrosis and Mitochondrial Dysfunction

**DOI:** 10.3390/biomedicines14051048

**Published:** 2026-05-05

**Authors:** Yingying Liao, Jie Xu, Yuheng Jiao, Xinxin Sun, Mingkui Gao, Yagang Ding, Dihui Cai, Yinyin Shen, Xiaohui Zhou, Wei Han

**Affiliations:** 1 Department of Cardiology, Shanghai East Hospital, School of Medicine, Tongji University, Shanghai 200092, China; 2332269@tongji.edu.cn (Y.L.); xujie8907@126.com (J.X.); drjyh97@163.com (Y.J.); caidihui@163.com (D.C.); yinyinshen2001@163.com (Y.S.); 2 Department of Cardiac Surgery, Shanghai East Hospital, School of Medicine, Tongji University, Shanghai 200092, China; sun_xinxin95@163.com (X.S.); gao1914615039@163.com (M.G.); sev7n520@163.com (Y.D.); 3 Research Center for Translational Medicine, Shanghai East Hospital, School of Medicine, Tongji University, Shanghai 200092, China; 4 State Key Laboratory of Cardiology and Medical Innovation Center, Shanghai East Hospital, School of Medicine, Tongji University, Shanghai 200092, China

**Keywords:** atrial fibrillation, humanin, MOTS-c, mitochondria

## Abstract

**Background:** Atrial fibrillation (AF) is a common clinical arrhythmia associated with mitochondrial dysfunction, oxidative stress, and atrial fibrosis. Mitochondrial-derived peptides (MDPs), including humanin (HN) and MOTS-c, exhibit cytoprotective properties, but their role in AF remains largely unknown. **Objective:** This study aimed to investigate the expression of HN and MOTS-c in AF patients and to evaluate their therapeutic potential and underlying mechanisms in an AngII-induced mouse model and primary cardiac cells. **Methods:** HN and MOTS-c expression in human atrial tissues was analyzed using public GEO data, immunohistochemistry, and immunofluorescence. Plasma levels were measured in a matched cohort (39 AF patients, 39 sinus rhythm controls). Murine AF models (male C57BL/6J mice, *n* = 36) and primary rat cardiomyocytes and fibroblasts were exposed to angiotensin II (AngII) with or without treatment with HNG (an HN analogue) or MOTS-c. **Results:** HN and MOTS-c were significantly downregulated in human AF atrial tissue, and their levels inversely correlated with fibrosis extent. Plasma MOTS-c was decreased in AF patients and inversely correlated with NT-proBNP. In vivo, HNG or MOTS-c treatment reduced AF inducibility and attenuated AngII-induced atrial fibrosis and hypertrophy. Peptide treatment was associated with improved mitochondrial ultrastructure, reduced mitochondrial fission proteins (Drp1, Fis1), and lower pro-inflammatory cytokines (IL-1β, IL-6) in mouse atria. In primary cardiomyocytes, both peptides mitigated AngII-induced oxidative stress. In fibroblasts, they directly inhibited AngII-induced activation, proliferation, and migration. Exploratory RNA-seq suggested that HNG predominantly affects cell adhesion pathways, while MOTS-c acts on metabolic processes. **Conclusions:** Downregulation of HN and MOTS-c in human AF is associated with disease severity. In murine models, HNG or MOTS-c administration attenuates atrial fibrosis and mitochondrial dysfunction and reduces AF inducibility. These findings suggest that MDPs may represent a novel therapeutic avenue for AF, although further validation with larger cohorts and mechanistic studies are required.

## 1. Introduction

Atrial fibrillation (AF), as the most prevalent clinical arrhythmia [1], often causes severe consequences such as stroke and heart failure (HF), thereby imposing a substantial societal burden [2]. The incidence of AF increases dramatically with age. Atrial electrical and structural remodeling are core pathophysiological mechanisms underlying the initiation and perpetuation of AF [3]. The current recommended therapies, including rate and rhythm control treatment and anticoagulation, fail to improve the outcomes and life quality of patients [4]. Even with extensive explorations, comprehensive mechanistic insight and targeted research to design precise interventions are still needed.

Atrial fibrosis is the central, dominant component of the structural remodeling process [3,5,6,7,8]. The hyperactivation, proliferation of fibroblasts, and excessive deposition of extracellular matrix, driven by factors like transforming growth factor-β and angiotensin II (AngII), are direct causes of fibrosis [3,8,9]. Previous studies confirmed the pivotal role of the renin–angiotensin–aldosterone system (RAAS) in this process. Inhibition of the angiotensin-converting enzyme suppresses atrial fibrosis and the development of persistent AF in an animal model [10]. Meanwhile, clinical studies reported a lower incidence of AF in selected patient populations treated with RAAS inhibitors [11]. Further supporting this, a subanalysis of the EMPHASIS-HF trial showed that treatment with the mineralocorticoid receptor antagonist eplerenone was associated with a lower incidence of new-onset AF compared to placebo, underscoring its potential for AF prevention [12]. Furthermore, the mineralocorticoid receptor blocker (MRB) eplerenone (EPL) reduced the AF burden by attenuating structural remodeling in experimental sheep AF models [13]. Despite these advances, therapeutic responses are often incomplete, prompting the search for complementary or alternative targets within the fibrotic cascade.

Recently, mitochondrial dysfunction has been increasingly recognized as a key contributor to the pathogenesis of AF [14,15,16,17]. The rapid atrial rate during AF leads to energy depletion and excessive production of reactive oxygen species (ROS), triggering oxidative stress, inflammatory responses, and apoptosis, which ultimately exacerbate electrical and structural deterioration [15,16,17,18]. Consequently, targeting mitochondrial function has emerged as a promising therapeutic direction [17].

Humanin (HN) and MOTS-c are recently identified micropeptides encoded by mitochondrial DNA [19,20]. Notably, bioinformatic analyses have identified 13 sequences (MT-RNR2L1–MT-RNR2L13) in the nuclear genome that are highly homologous to the mitochondrial MT-RNR2 gene, at least 10 of which can be transcribed into mRNA and potentially translated into HN-like peptides [21]. Preclinical studies have demonstrated their broad cytoprotective capacities. HN protects endothelial cells from oxidized low-density lipoprotein (ox-LDL)-induced oxidative stress and apoptosis [22]. In aged mice, administration of HNG ameliorates myocardial fibrosis and oxidative stress [23]. In cerebral ischemia models, HNG reduces infarction area and pro-inflammatory cytokine expression [24]. Similarly, MOTS-c mitigates cardiac dysfunction, inflammation, and oxidative stress in transverse aortic constriction (TAC)-induced murine HF models [25] and enhances cardiac mechanical efficiency in exercised rats [26]. Recent study revealed the protective effects of MOTS-c in rat models of acute lung injury following cardiac ischemia [27]. However, whether and how humanin (HN) and MOTS-c attenuate AF remains elusive.

Despite these findings in other cardiovascular conditions, the expression, functional significance, and therapeutic potential of HN and MOTS-c in the specific context of AF remain entirely unexplored. This gap is particularly relevant given that AF shares pathogenic mechanisms such as fibrosis, oxidative stress, and mitochondrial dysfunction—all known to be modulated by these peptides. Therefore, the novelty of this study lies in being the first to investigate HN and MOTS-c in AF.

We hypothesized that HN and MOTS-c are downregulated in AF, and that exogenous supplementation with HNG or MOTS-c could ameliorate AF by improving mitochondrial function and suppressing fibrosis. To test this hypothesis, we employed a multi-tiered strategy: (1) we measured HN and MOTS-c expression in human AF atrial tissues and plasma; (2) we evaluated the therapeutic effects of HNG and MOTS-c in an AngII-induced mouse AF model; and (3) we explored the cellular mechanisms in primary cardiomyocytes and fibroblasts.

## 2. Material and Methods

### 2.1. Human Samples

Study design and participants

This study included two independent human cohorts: a plasma cohort for peptide level measurement and a tissue cohort for histological analysis. Participants in both cohorts were consecutively enrolled (not randomly selected) as they presented for routine health examinations or cardiac surgery.

Plasma cohort

Recruitment and enrollment: Elderly residents (age > 60 years) undergoing routine health examinations at the Beicai Community Health Center in Shanghai were recruited.

Diagnostic criteria: Participants were classified into the atrial fibrillation (AF) group if they had both a self-reported history of AF from a questionnaire and AF documented on a standard 12-lead electrocardiogram (ECG) obtained during the examination. Participants were classified into the sinus rhythm (SR) group if they had no self-reported history of AF and a sinus rhythm ECG. Because AF diagnosis relied solely on a single ECG and self-report, reliable classification of paroxysmal versus persistent AF was not possible for this community-based cohort.

Matching: For each identified AF patient, one SR control subject was selected using 1:1 propensity score matching based on age, sex, hypertension, diabetes mellitus, and left ventricular ejection fraction (LVEF) measured by echocardiography.

Sample size: Initially, 40 matched pairs were enrolled. One plasma sample from an AF patient was found empty during processing, leading to the exclusion of that pair. Consequently, 39 matched pairs (n = 39 per group) were included in the final analysis.

Exclusion criteria: Participants with any of the following were excluded: (1) history of myocardial infarction or catheter ablation for AF; (2) chronic inflammatory diseases (determined by medical history and white blood cell count); (3) malignancy; (4) severe hepatic or renal dysfunction; (5) psychiatric disorders compromising reliable data collection.

Data collection: Clinical data, including medical history and medication use, were obtained through questionnaires administered at the time of the health examination.

Laboratory measurements: Plasma levels of humanin and MOTS-c were measured using commercial ELISA kits (EH9261 for humanin, EH5056 for MOTS-c, FineTest, Wuhan, China) according to the manufacturer’s instructions [28].

Ethics and consent: All participants provided written informed consent. The study was approved by the Ethics Committee of Shanghai East Hospital and registered at ClinicalTrials.gov (NCT06673615). Baseline characteristics are shown in Table S1.

Tissue cohort

Recruitment and enrollment: Human right atrial appendage tissues were obtained from patients (>18 years) undergoing elective open-heart surgery at Shanghai East Hospital.

Diagnostic categorization: Patients were classified into AF (including paroxysmal and persistent AF) or SR group based on clinical chart diagnoses.

Sample size: Atrial tissues from 13 individuals (6 SR, 7 AF) were used for histological and immunohistochemical analyses.

Exclusion criteria: Patients with a diagnosis of post-operative AF, autoimmune disorders, or systemic inflammatory conditions were excluded.

Data collection: Clinical data were retrieved from electronic medical records.

Histopathological analysis: Tissues were processed for immunohistochemistry and immunofluorescence as described below.

Ethics and consent: Written informed consent was obtained from all patients before surgery. The study protocol was approved by the Ethics Committee of Shanghai East Hospital (approval number: 2025YS-159). Baseline characteristics of these patients are listed in Table S2.

### 2.2. Experimental Animals

All animal procedures complied with the guidelines of the Chinese National Institutes of Health and Animal Care, and received approval from the Animal Care Ethics Committee of Tongji University School of Medicine (approval No. TJBB09125103). Animals were maintained under controlled temperature, humidity, and a 12 h light/dark cycle. All animals that finished the study protocol were included in the final data analysis, with no post-allocation exclusions. The study established humane exclusion criteria, including severe illness, inability to consume food or water, or weight loss over 20%. None of the animals met these criteria during the course of the experiment.

### 2.3. AngII-Induced Atrial Fibrillation Murine Models with Peptide Treatment

Thirty-two male C57BL/6J mice (7 weeks old, 20–25 g) were purchased from GemPharmatech Co., Ltd. (Nanjing, China; License No. SCXK (Su) 2023-0009). The animals were housed under specific-pathogen-free (SPF) conditions in the Animal Facility of Shanghai East Hospital, Tongji University School of Medicine. Following a one-week acclimatization period (at 8 weeks of age), the mice were randomly assigned to four experimental groups using a computer-generated random number sequence: Control group (n = 9), AngII group (n = 8), AngII + HNG group (n = 8), and AngII + MOTS-c group (n = 7). Mice were infused subcutaneously with saline or AngII (T8560, TargetMol, USA) at a dose of 3 mg/kg/d via osmotic mini-pumps (2004w, Shenzhen RWD Life Science Co., Ltd., Shenzhen, China) for 3 weeks as described previously [29]. Concurrently, mice in the treatment groups received intraperitoneal injections of either HNG (HY-P3993A, MCE, Monmouth Junction, NJ, USA) at 4 mg/kg/d or MOTS-c (TP2312, TargetMol, Boston, MA, USA) at 3 mg/kg/d, five days per week, for the same 3-week duration, with reference to previous studies [23,30,31]. Control animals received same dose of sterile PBS vehicle injections on the same schedule.

### 2.4. Isolation and Culture of Primary Rat Atrial Cells

Primary rat atrial myocytes (NRAMs) and fibroblasts were isolated from the atrial tissue of 1–3-day-old Sprague-Dawley rats using a differential adhesion protocol, as previously described with modifications [32]. Briefly, the hearts were rapidly excised, and the atria were separated, minced into 1–2 mm^3^ fragments, and subjected to sequential digestion with 0.25% trypsin at 37 °C. Dissociated cells were collected by centrifugation at 1500× *g* for 5 min and resuspended in DMEM (Biological Industries, Kibbut z Beit Haemek, Israel) supplemented with 10% fetal bovine serum (FBS, Biological Industries) and 1% penicillin/streptomycin (Beyotime, Shanghai, China). To separate cell types, the total cell suspension was plated in a culture dish and incubated for 45 min, allowing fibroblasts to adhere. The supernatant containing the non-adherent cardiac myocytes was then gently transferred and seeded into 6-well plates at a density of 3 × 10^5^ cells per well in DMEM containing 10% FBS for subsequent experiments. The adherent fibroblasts were cultured in the original dish for 48 h, after which they were trypsinized, counted, and seeded into 6-well plates at a density of 3 × 10^5^ cells per well in DMEM containing 10% FBS for expansion prior to experiments.

### 2.5. Drug Treatment Protocol

For in vitro experiments, both cell types were pretreated with either 10 μmol/L HNG (HY-P3993A, MCE) or 5 μmol/L MOTS-c(TP2312, TargetMol) for 1 h, followed by co-treatment with 1 μmol/L AngII (T8560, TargetMol) for 48 h. During the 48 h treatment period, fibroblasts were maintained in DMEM containing 2% FBS, while cardiomyocytes were kept in DMEM with 10% FBS. All cells were cultured at 37 °C in a humidified atmosphere of 5% CO_2_.

### 2.6. Histology and Immunohistochemistry

Atrial tissue samples from human and mouse sources were fixed in paraffin and sectioned at 5–7 μm thickness. Deparaffinization was performed using xylene, followed by rehydration through a graded alcohol series. Sections were then stained with hematoxylin solution, rinsed under running water, and differentiated with 1% acid alcohol. To evaluate atrial interstitial fibrosis, Masson’s trichrome and Picrosirius red staining were employed. For immunofluorescence analysis, 8 μm-thick paraffin sections were subjected to antigen retrieval with citrate buffer and incubated with primary antibodies overnight at 4 °C. Subsequently, sections were labeled with Alexa Fluor-conjugated secondary antibodies. Nuclear counterstaining was performed using DAPI, while the extracellular matrix was visualized with wheat germ agglutinin (WGA, Thermo Fisher Scientific, Waltham, MA, USA). Representative images were obtained using a Zeiss microscope, and fibrotic regions or antibody-positive areas were quantified with ImageJ software (v1.8.0).

### 2.7. Measurement of Intracellular Reactive Oxygen Species (ROS)

Intracellular ROS levels were assessed using dihydroethidium (DHE) staining combined with both fluorescence microscopy and flow cytometry [32,33]. For both approaches, cells were incubated with 4 µM DHE (Thermo Fisher Scientific) at 37 °C for 20 min in the dark. For microscopic analysis, cells were washed with PBS after incubation. DHE fluorescence (indicative of superoxide oxidation) was visualized with Leica DM4B fluorescence microscope (excitation/emission: ~550/570 nm) and quantified with ImageJ software (v1.8.0). For flow cytometric analysis, cells were harvested after DHE staining, washed twice with PBS by centrifugation at 800 rpm for 5 min at room temperature, and then resuspended in 200 µL of PBS. The fluorescence intensity of oxidized DHE was immediately measured on a flow cytometer (Beckman cytoFLEX) using the PE channel (excitation: 488 nm; emission: 585 nm) and analyzed with FlowJo software (v10.8.1).

### 2.8. Western Blot Analysis

Protein lysates were prepared from cultured cells and atrial tissues using RIPA buffer (Beyotime). Equal amounts of protein (20–40 μg) were resolved via SDS-PAGE and electrotransferred onto PVDF membranes (Millipore, Burlington, MA, USA). The membrane was blocked for 1 h at room temperature, then incubated with primary antibody overnight at 4 °C. After incubation with appropriate secondary antibodies, protein signals were detected using enhanced chemiluminescence reagents (Epizyme Biotech, Shanghai, China) and imaged with a ChemiDoc MP system (Bio-Rad Laboratories, Hercules, CA, USA). The primary antibodies used were as follows: Collagen I (66761-1-1g, proteintech, China), α-SMA (A17910, ABclonal, Wuhan, China), Drp1 (A21968, ABclonal), Fis1 (32525, cell signaling, Danvers, MA, USA), GAPDH (A19056, ABclonal), β-actin (AC026, ABclonal) and horseradish peroxidase-labeled secondary antibodies (Beyotime).

### 2.9. Quantitative Real-Time Polymerase Chain Reaction

Total RNA was isolated from cultured cells and atrial tissue samples employing the SteadyPure Quick RNA Extraction Kit (Accurate Biotechnology, Hunan, China). Complementary DNA was synthesized using the HiScript III RT SuperMix for qPCR (Vazyme, Nanjing, China). All subsequent amplifications were conducted using a QuantStudio™ 6 Flex Real-Time PCR Systems (Thermo Fisher Scientific) and ChamQ Universal SYBR qPCR Master Mix (Vazyme). Relative mRNA levels were calculated by the 2^−ΔΔCt^ method, with β-actin serving as the internal reference gene. Primer sequences are provided in Table S3.

### 2.10. Superoxide Dismutase (SOD)

Cellular samples from different experimental groups were analyzed to determine superoxide dismutase (SOD) activity levels. The measurements were performed with commercial SOD assay kits (Beyotime), following the standardized protocols provided by the manufacturer.

### 2.11. Transesophageal Programmed Electrical Stimulation

Transesophageal programmed electrical stimulation was performed to assess atrial fibrillation (AF) inducibility [34,35]. Mice were anesthetized via intraperitoneal administration of tribromoethanol (2.5%, 0.02 mL/g; Aibei Biotechnology Co., Ltd., Nanjing, China). Under stable anesthesia, a 1.1F octapolar electrophysiology catheter (Millar Instruments, Houston, TX, USA) was advanced transesophageally and positioned at the level of the left atrium for subsequent programmed stimulation. Each mouse underwent the same atrial burst-pacing protocol three times. AF was defined as a period of rapid, irregular atrial activity lasting >1 s. Mice were classified as AF-positive when atrial fibrillation was elicited in a minimum of two out of three pacing trials. The incidence of inducible AF was calculated as the percentage of AF-positive mice in each group. To ensure animal welfare, any AF episode was terminated by gentle tactile stimulation if it exceeded 10 min. Electrophysiological signals were recorded and analyzed using LabChart Pro software (v7.3, ADInstruments, Dunedin, New Zealand). Throughout the procedure and analysis, the operator was blinded to the treatment groups of the mice.

### 2.12. Cell Proliferation Assay (CCK-8)

Cell proliferation was assessed using the Cell Counting Kit-8 (CCK-8, Dojindo, Kumamoto, Japan) according to the manufacturer’s instructions. Primary rat atrial fibroblasts were seeded into 96-well plates and cultured overnight. After the indicated treatments for 48 h, 10 µL of CCK-8 solution was added to each well and incubated for an additional 1 h at 37 °C. Absorbance was measured at 450 nm using a microplate reader.

### 2.13. Wound Healing Assay

Cell migration was evaluated using a wound healing assay. Primary rat atrial fibroblasts were seeded into 6-well plates and cultured until reaching approximately 90% confluence. A linear scratch wound was created across the cell monolayer using a sterile 200 µL pipette tip. Wound closure was photographed at 0, 24, and 48 h using an inverted microscope. The wound area was quantified using ImageJ software, and the migration rate was calculated as the percentage of wound closure relative to the 0 h time point.

### 2.14. Bioinformatic Analysis of Spatial Transcriptomic Data

The spatial transcriptomic dataset GSE261363 was downloaded from the Gene Expression Omnibus (GEO) database (http://www.ncbi.nlm.nih.gov/geo/, accessed on 14 October 2024). We processed and normalized the dataset using the GEOquery package (v2.66.0) in R [36]. To identify differentially expressed genes (DEGs), the limma package (version 4.4.1) was applied [37]. Genes with *p*-values < 0.05 and log-fold changes > 1 were considered significantly differentially expressed. Volcano plots and heatmaps were generated using R packages (‘ggplot2′v3.5.1 and ‘pheatmap’v1.0.12) [38]. For each donor, six distinct tissue regions were analyzed, resulting in 12 columns per group in the heatmap.

### 2.15. RNA Sequencing of Primary Cardiac Fibroblasts

Primary rat atrial fibroblasts were treated with AngII alone, AngII combined with HNG, or AngII combined with MOTS-c for 48 h, with four biological replicates per group. Total RNA was extracted using TRIzol reagent (Invitrogen, Carlsbad, CA, USA) following the manufacturer’s instructions. RNA integrity was assessed, and qualified RNA samples were used for library preparation. Sequencing libraries were constructed using the Hieff NGS Ultima Dual-mode mRNA Library Prep Kit (Yeasen Biotechnology, Shanghai, China) according to the manufacturer’s protocol. The prepared libraries were sequenced on a DNBSEQ platform (BGI, Shenzhen, China) to generate paired-end reads. The raw RNA-seq data have been deposited in the Gene Expression Omnibus (GEO) database under accession number GSE322635. Principal component analysis (PCA) and Gene Ontology (GO) enrichment analysis were performed using the online analysis platform (https://cloud.tsingke.com.cn, accessed on 13 July 2025).

### 2.16. Outcome Measures

The study evaluated outcomes encompassing electrophysiological, histopathological, molecular, and cellular parameters. Electrophysiological assessments focused on atrial fibrillation (AF) inducibility using transesophageal burst pacing, where AF was defined as sustained irregular atrial activity exceeding 1 s. Histopathological endpoints included atrial fibrosis quantified by Picrosirius red and Masson’s trichrome staining, cardiomyocyte hypertrophy determined by wheat germ agglutinin (WGA) staining, and mitochondrial morphology examined via transmission electron microscopy. Molecular measurements consisted of protein and mRNA expression levels of Humanin (HN) and MOTS-c in human atrial tissues and mouse atria, along with markers of mitochondrial fission (Drp1, Fis1), pro-inflammatory cytokines (IL-1β, IL-6), myocardial stress (Nppa, Acta1, Myh7, Sesn2), and fibroblast activation (α-SMA, Col1a1). Cellular endpoints included reactive oxygen species (ROS) generation, superoxide dismutase (SOD) activity in primary cardiomyocytes, and fibroblast proliferation (CCK-8 assay) and migration (wound healing assay).

No formal sample-size calculation was performed because this was an exploratory study. As this study was designed as an exploratory mechanistic investigation, a single primary outcome measure was not predefined for sample size calculation. Rather, treatment effects were assessed based on the consistency of findings across multiple independent outcomes spanning electrophysiological, histopathological, molecular, and cellular domains.

### 2.17. Data Analysis and Statistical Methods

Statistical analyses were performed with GraphPad Prism software (version 8.0). Data for continuous variables are expressed as mean ± standard deviation (SD). The normality of data distribution for each dataset was evaluated using the Shapiro–Wilk test. For comparisons between two groups of normally distributed data, an unpaired two-tailed Student’s t-test was applied. Comparisons among more than two groups of normally distributed data were conducted by one-way analysis of variance (ANOVA), followed by Tukey’s post hoc analysis for multiple comparisons. Non-normally distributed data were analyzed using the Mann–Whitney U test (two groups) or the Kruskal–Wallis test with Dunn’s post hoc correction (more than two groups). Associations between continuous variables were assessed using Pearson’s correlation analysis for normally distributed data or Spearman’s rank correlation for non-normally distributed data. For categorical variables, the χ^2^ test was used to compare observed frequencies with expected frequencies under the null hypothesis of no association. When expected cell frequencies were less than 5, Fisher’s exact test was applied instead. A two-sided *p* value of less than 0.05 was considered statistically significant.

## 3. Results

### 3.1. Both Expressions of HN and MOTS-c Are Downregulated in Human Atrial Fibrillation Tissue and Negatively Correlate with Fibrosis

**Figure 1:**
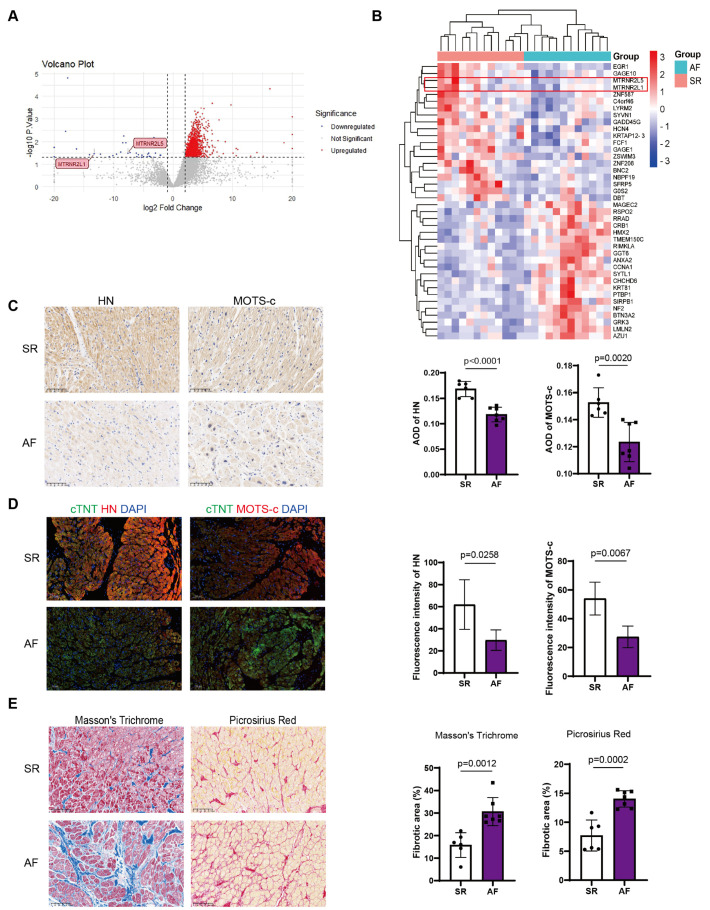
**Both HN and MOTS-c levels decreased in atrial tissues of atrial fibrillation (AF).** (**A**) Reanalysis of spatial transcriptomic data (GEO dataset GSE261363) showing downregulated expression of the nuclear-encoded humanin genes (MTRNR2L1, MTRNR2L5) in atrial myocardial tissue from donors with atrial fibrillation (AF, n = 2 donors) compared to those with sinus rhythm (SR, n = 2 donors). (**B**) Heatmap generated from the same dataset (GSE261363), displaying the expression patterns of the top 20 differentially expressed genes. Six distinct tissue regions were analyzed per donor, resulting in 12 columns per group (SR, AF). (**C**) Representative immunohistochemical (IHC) staining and quantification of HN and MOTS-c protein expression in human right atrial appendage tissues from patients with SR (n = 6) and AF (n = 7). Scale bars, 100 μm. (**D**) Representative immunofluorescence (IF) staining and quantification of HN and MOTS-c in human atrial tissues from SR (n = 6) and AF (n = 7) patients. Scale bars, 100 μm. (**E**) Representative Masson’s trichrome staining and Picrosirius red staining of human atrial tissues, showing increased collagen deposition in AF (n = 6 or 7). Scale bars, 100 μm. Data are shown as mean ± SD. Data in (**C**,**D**) were analyzed via the Student *t*-tests. Data of Masson’s trichrome staining in E were analyzed via the Mann–Whitney U tests. Data of Picrosirius red staining in (**E**) was analyzed via the Student *t*-tests.

Spatial transcriptomic data from the GEO database (GSE261363) revealed significant downregulation of nuclear-encoded HN genes in AF atrial myocardial tissues compared to SR controls (Figure 1A,B). Consistent with the transcriptomic findings, IHC and IF staining further confirmed the substantial reductions of both HN (Figure 1C,D) and MOTS-c (Figure 1C,D) protein levels in atrial appendage of AF patients, compared to the SR control.

**Figure 2:**
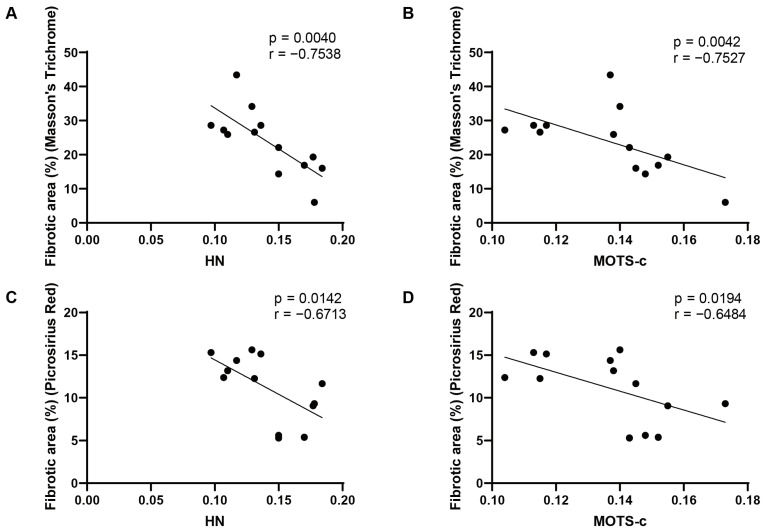
**Correlations of HN and MOTS-C expression with atrial fibrosis degree in human atrial tissues.** Correlation analysis between quantitative fibrosis area from Masson’s staining (**A**,**B**) and Picrosirius red staining (**C**,**D**) and the protein expression levels of HN (**A**,**C**) or MOTS-c (**B**,**D**) (quantified from IHC) (n = 13). Spearman rank correlation coefficient quantifies these correlations.

Next, Masson’s trichrome and Picrosirius red staining showed a marked increase of collagen deposition in the atria of AF patients (Figure 1E). Importantly, quantitative correlation analysis demonstrated a significant negative relationship between the levels of both HN and MOTS-c and the degree of fibrosis quantified by both Masson’s trichrome (Figure 2A,B) and Picrosirius red staining (Figure 2C,D). These results indicated that the downregulation of mitochondrial peptides HN and MOTS-c in the human AF atrium is closely associated with the progression or severity of atrial fibrosis.

### 3.2. Plasma MOTS-c Level Decreased in AF Patients and Inversely Correlates with NT-proBNP Level (N-Terminal pro-B-Type Natriuretic Peptide)

**Figure 3:**
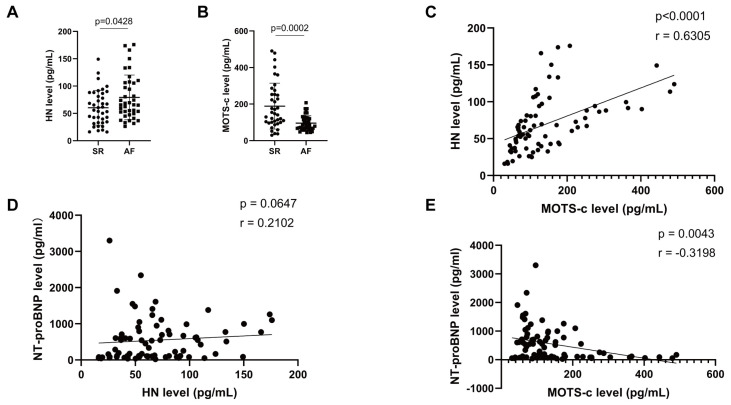
**Plasma MOTS-c level is downregulated in patients with AF and inversely associated with NT-proBNP production.** (**A**,**B**) ELISA was performed to measure plasma HN (**A**) and MOTS-c (**B**) concentrations in patients with atrial fibrillation (AF; n = 39) and control subjects with sinus rhythm (SR; n = 39). (**C**) Spearman correlation analysis between plasma HN and MOTS-c levels in patients above (n = 78). (**D**,**E**) Spearman correlation analysis of plasma HN (**D**) or MOTS-c (**E**) levels with NT-proBNP levels in patients above (n = 78). Data are shown as mean ± SD. Data in (**A**,**B**) were analyzed via the Mann–Whitney U tests.

We also measured the levels of these peptides in plasma. As shown by Figure 3B, MOTS-c concentration was significantly lower in AF patients than that in SR controls (Figure 3B). Interestingly, plasma HN levels were slightly elevated in AF patients (Figure 3A). A positive correlation was observed between plasma levels of HN and MOTS-c (Figure 3C). Furthermore, MOTS-c expressions exhibited a significant inverse correlation with NT-proBNP levels (Figure 3E), while no significant correlation was found between HN and NT-proBNP (Figure 3D). These results suggest that plasma MOTS-c, but not HN, may reflect AF-related pathological stress, pending validation as a biomarker.

### 3.3. Administration of HNG or MOTS-c Attenuates AngII-Induced Atrial Fibrillation Susceptibility and Structural Remodeling in Mice

**Figure 4:**
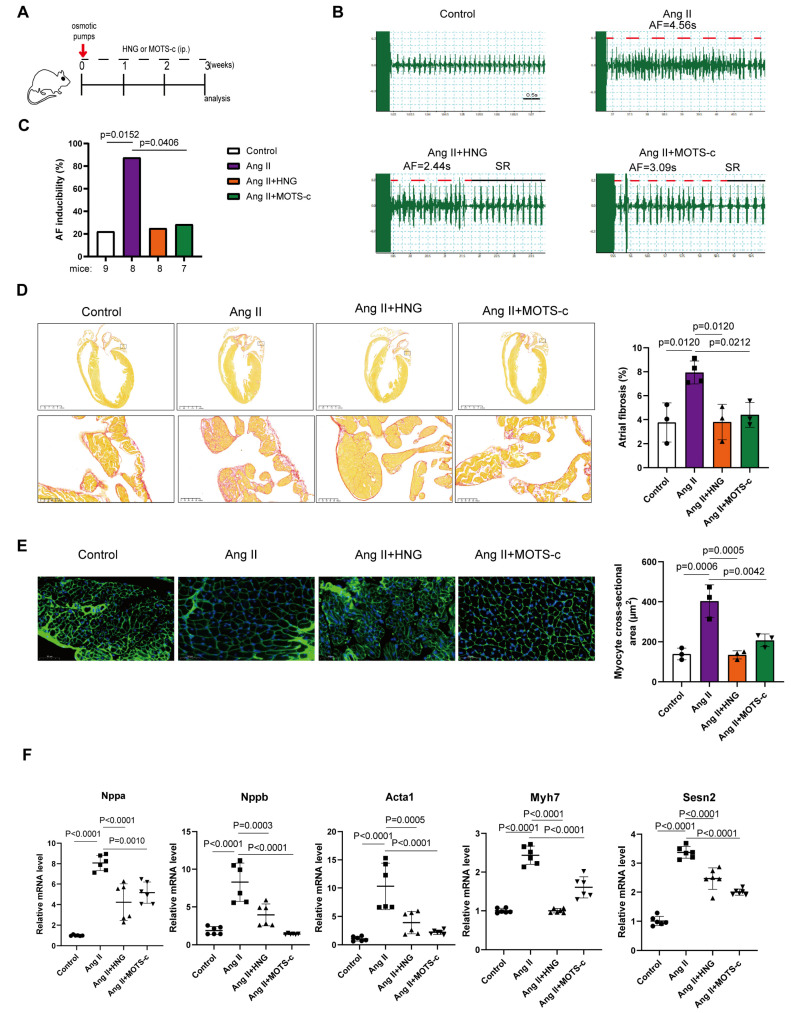
**HNG and MOTS-c treatment attenuates AngII-induced atrial fibrillation (AF) susceptibility and structural remodeling in mice.** (**A**) Schematic timeline of the experimental protocol. (**B**) Representative electrocardiogram (ECG) tracings of burst pacing-induced AF. (**C**) Bar graph summarizing the incidence of inducible AF in each group. (**D**) Representative images of Picrosirius red-stained whole heart sections from each experimental group, alongside quantification of fibrotic area (red) in both atrial chambers. Scale bars, 2.5 mm or 100 μm. (**E**) Representative images of wheat germ agglutinin (WGA) staining of the mouse atria from the indicated groups, showing cardiomyocyte cross-sectional area. A total of 250 cells per mouse were measured. Scale bar, 50 μm. (**F**) mRNA expression levels of myocardial stress markers (Nppa, Nppb, Acta1, Myh7, Sesn2) in mouse atrial tissues. n = 3 to 9 mice per group for all experiments. Data are shown as mean ± SD. Data points in the graphs represent biological replicates. Data in (**C**) were analyzed via χ^2^ test (AF inducibility). Data in (**D**–**F**) were analyzed via one-way ANOVA, followed by Tukey’s post hoc analysis.

To determine the therapeutic potential of these peptides, we employed an AngII-infused murine AF model (experimental timeline, Figure 4A). (Gly14)-Humanin (HNG), a synthetic and more potent analogue of HN with a glycine-for-serine substitution at position 14 was utilized [39]. AngII treatment significantly increased AF inducibility upon transesophageal electrical pacing compared to the control group, as evidenced by representative electrocardiograms (Figure 4B) and summary data (Figure 4C). This pro-arrhythmic effect was markedly attenuated by concurrent treatment with either HNG (a potent HN analogue) or MOTS-c (Figure 4B,C).

Histological examination revealed that AngII infusion induced pronounced atrial fibrosis, which was ameliorated by both HNG and MOTS-c treatments (Figure 4D). Furthermore, Wheat Germ Agglutinin (WGA) staining showed that cardiomyocyte hypertrophy induced by AngII was also suppressed by either peptide administration (Figure 4E). At the molecular level, the upregulated mRNA expressions of myocardial stress markers, including Nppa, Nppb, Acta1, Myh7 [40], and Sesn2 [41] in AngII-treated mice, were significantly blunted in the HNG and MOTS-c treatment groups (Figure 4F). These data demonstrated that inter peritoneal administration of HNG and MOTS-c can effectively protect the AngII-treated mice from AF susceptibility as well as mitigate the cardiac structural remodeling.

### 3.4. HNG and MOTS-c Preserve Mitochondrial Integrity and Alleviate Oxidative Stress and Inflammation

**Figure 5:**
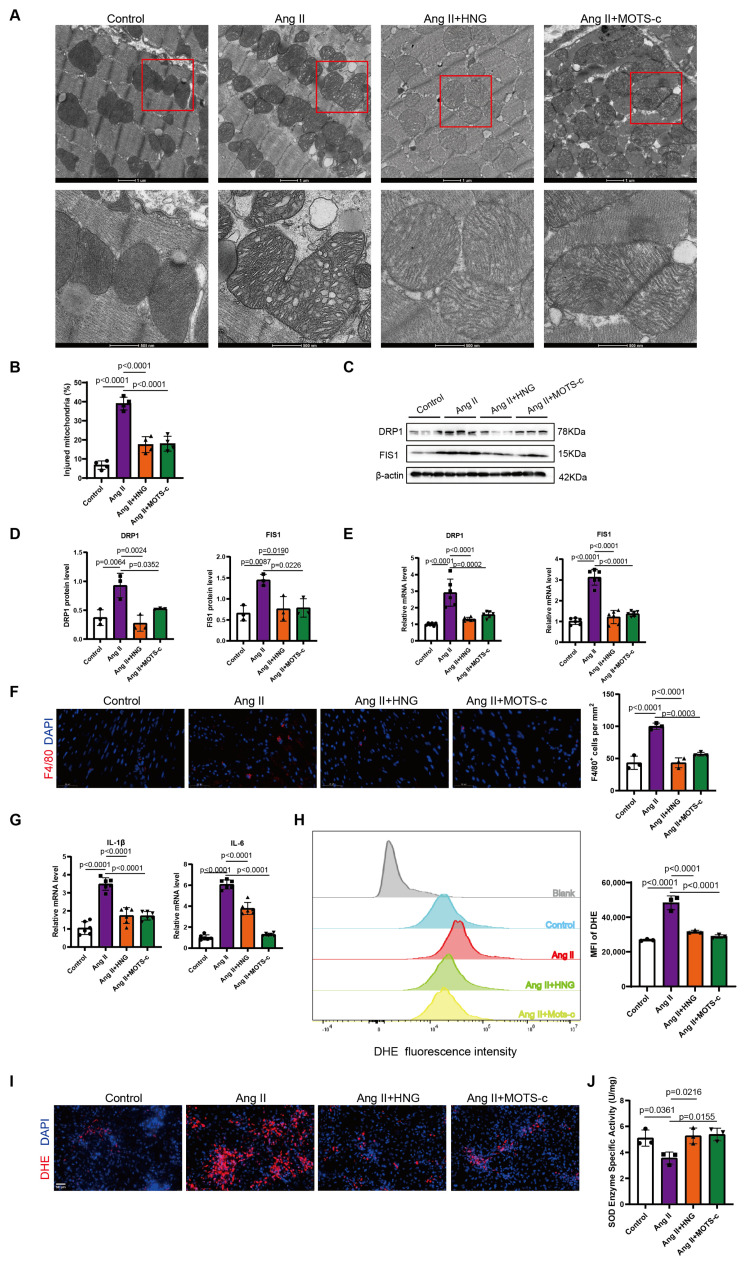
**HNG and MOTS-c preserve mitochondrial integrity and alleviate inflammation and oxidative stress in mouse atria and primary cardiomyocytes.** (**A**) Representative transmission electron micrographs of mitochondria in mouse atrial from the indicated groups. Scalebar, 1 μm or 500 nm. (**B**) Quantification of injured mitochondria in mouse atria. At least 50 mitochondria per animal were analyzed. (**C**–**E**) Representative Western blot (**C**) with quantitative analysis (**D**) and mRNA (**E**) of mitochondrial fission proteins (Drp1, Fis1) in mouse atrial tissues. (**F**) Representative immunofluorescence staining and quantification of F4/80 (macrophage marker) in mouse atrial tissues. Scale bar, 50 μm. (**G**) mRNA expression levels of pro-inflammatory cytokines (IL-1β, IL-6) in mouse atrial tissues. (**H**–**J**) In vitro studies on primary rat atrial cardiomyocytes. (**H**) Flow cytometry analysis of reactive oxygen species (ROS) using DHE probe. (**I**) Representative DHE fluorescence staining (red) and DAPI (blue). Scale bar, 50 μm. (**J**) Superoxide dismutase (SOD) activity assay. n = 3–6 per group for in vivo, n refers to the number of mice. n = 3 independent experiments for in vitro, n refers to independent biological replicates. Data are shown as mean ± SD. Data points in the graphs represent biological replicates. Data in (**B)** to (**J**) were analyzed via one-way ANOVA, followed by Tukey’s post hoc analysis.

We next explored the mechanisms underlying the protective effects of these two peptides. Electron microscopy revealed severe mitochondrial ultrastructural damage in the atria of AngII-treated mice, which was notably prevented by HNG and MOTS-c treatment (Figure 5A,B). In addition, AngII infusion led to increased mRNA and protein expression of mitochondrial fission proteins Drp1 and Fis1, indicating aberrant mitochondrial dynamics. This effect was reversed by both peptide treatments (Figure 5C–E). Concurrently, the elevated mRNA levels of pro-inflammatory cytokines IL-1β and IL-6 in the AngII group were reduced following HNG and MOTS-c administration (Figure 5G). Immunofluorescence staining and quantification of F4/80 confirmed a reduction of macrophage infiltration into the heart of the treatment groups compared to the AF heart (Figure 5F).

In primary rat atrial cardiomyocytes, AngII challenge for 48 h significantly increased oxidative stress, as detected by elevated ROS levels in flow cytometry and DHE staining, and decreased SOD activity. Pre-treatment with either HNG or MOTS-c effectively mitigated these changes (Figure 5H–J). Collectively, these findings indicated that the protective efficacy of HNG and MOTS-c in AF models may attribute to their function to preserve mitochondrial integrity, suppress inflammatory responses, and reduce oxidative stress in the heart.

### 3.5. HNG and MOTS-c Inhibit AngII-Induced Activation, Proliferation, and Migration of Cardiac Fibroblasts

**Figure 6:**
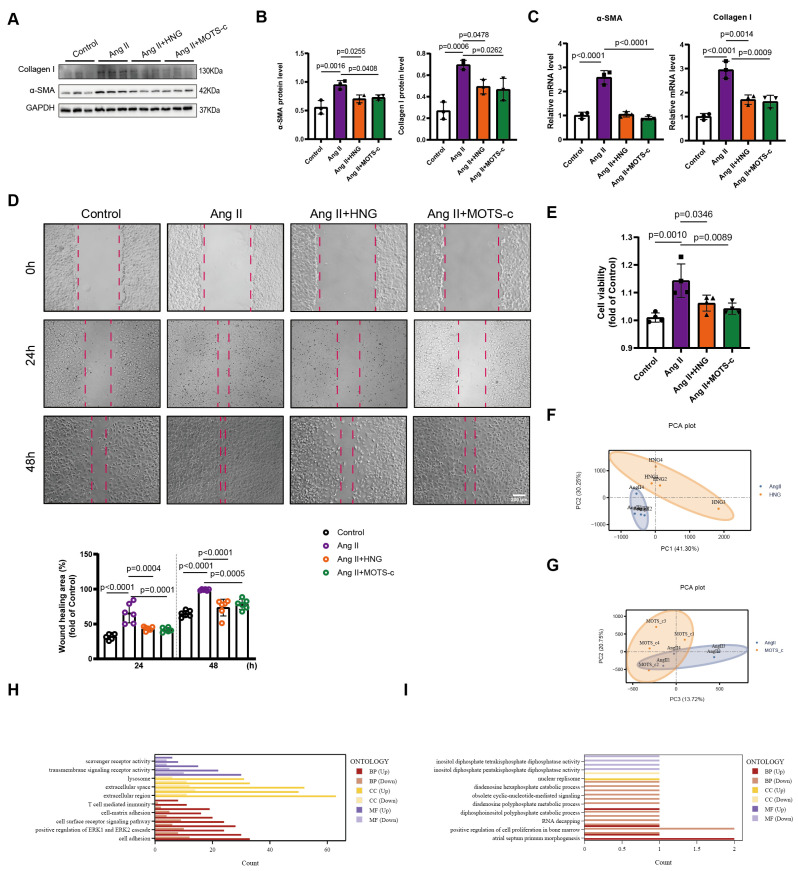
**HNG and MOTS-c inhibit AngII-induced activation, proliferation, and migration of primary cardiac fibroblasts.** All experiments were performed on primary rat atrial fibroblasts. (**A**–**C**) Representative Western blot (**A**) with quantitative analysis (**B**) and mRNA expression (**C**) of fibroblast activation markers (α-SMA, Col1a1). (**D**) Cell migration assessed by wound healing assay. Representative images at 0 h and 48 h are shown (scale bar, 200 μm), with quantitative analysis of migration rate. (**E**) Cell proliferation assessed by CCK-8 assay. n = 3–6 independent experiments for in vitro. Data points in the graphs represent biological replicates. Data in B to E were analyzed via one-way ANOVA, followed by Tukey’s post hoc analysis. (**F**,**G**) RNA sequencing analysis of primary cardiac fibroblasts. Principal component analysis (PCA) plots of global gene expression profiles for cells treated with AngII versus AngII + HNG (**F**) and AngII versus AngII+MOTS-c (**G**) (n = 4 independent samples per group). (**H**,**I**) Gene Ontology (GO) enrichment analysis of biological processes. Shown are the top enriched terms for differentially expressed genes identified between AngII-treated fibroblasts and the AngII + HNG group (**H**) (filtered by *p* < 0.05), and between AngII-treated fibroblasts and the AngII + MOTS-c group (**I**) (filtered by *p* < 0.05).

Since atrial fibrosis is a hallmark of AF, we then investigated the direct effects of the peptides on cardiac fibroblasts. In primary rat cardiac fibroblasts, AngII stimulation significantly upregulated the mRNA and protein expression of activation markers α-SMA and Col1a1 (Figure 6A–C). This profibrotic activation response was suppressed by pre-treatment with HNG or MOTS-c. CCK-8 assays demonstrated that AngII-enhanced fibroblast proliferation was also inhibited by both peptides (Figure 6E). Finally, a wound healing assay showed that the increased migratory capacity of fibroblasts induced by AngII was markedly reduced by addition of HNG and MOTS-c (Figure 6D). These results provided direct evidence that HNG and MOTS-c exert potent anti-fibrotic effects by attenuating the pro-fibrotic phenotype of cardiac fibroblasts.

To gain a comprehensive understanding of how HNG and MOTS-c modulate fibroblast phenotype at the transcriptional level, we performed RNA sequencing on primary cardiac fibroblasts treated with AngII, AngII+HNG, or AngII+MOTS-c. Principal component analysis (PCA) of the global gene expression data revealed a clear separation between AngII-treated fibroblasts and those co-treated with either HNG (Figure 6F) or MOTS-c (Figure 6G), indicating that both peptides profoundly altered the AngII-induced transcriptional landscape.

Gene Ontology (GO) enrichment analysis of the differentially expressed genes identified distinct biological processes influenced by each peptide. Compared to the AngII group, HNG treatment predominantly reversed genes associated with signal transduction, immune response, and cell-substrate adhesion (Figure 6H). In contrast, MOTS-c treatment primarily normalized the expression of genes involved in various metabolic processes (Figure 6I). To validate the RNA-seq findings, we performed qPCR on selected upregulated and downregulated genes in independent fibroblast samples (n = 4 per group), which confirmed the direction of expression changes for both HNG and MOTS-c (Figure S1). These transcriptomic findings are hypothesis-generating and suggest distinct biological programs.

## 4. Discussion

In this study, we provide the first evidence linking the mitochondrial-derived peptides HN and MOTS-c to atrial fibrillation (AF). Our principal findings are threefold. First, HN and MOTS-c are significantly downregulated in human AF atrial tissue, and their levels inversely correlate with the extent of fibrosis. Second, plasma MOTS-c is decreased in AF patients and inversely associated with NT-proBNP, whereas plasma HN shows a slight elevation. Third, in an AngII-induced mouse model, treatment with HNG (an HN analogue) or MOTS-c reduces AF inducibility, attenuates atrial fibrosis and hypertrophy, and ameliorates mitochondrial dysfunction, oxidative stress, and inflammation. Additionally, both peptides directly suppress AngII-induced activation, proliferation, and migration of cardiac fibroblasts in vitro. Collectively, these findings suggest that restoring HN/MOTS-c levels may represent a novel therapeutic strategy for AF, although further validation is required.

Previous observations suggested a complex profile of HN and MOTS-c in peripheral blood and tissues in different clinical settings. It was reported that plasma HN levels were lower in patients with angina and myocardial infarction [42]. Conversely, in a large aging cohort (693 individuals, 21–113 years), plasma HN increases with age but paradoxically associates with worse metabolic and functional parameters in the elderly [43]. In chronic kidney disease, circulating HN is elevated while its muscle expression is reduced, and circulating HN correlates positively with inflammatory mediators; meanwhile, MOTS-c level decreased in both compartments [44]. Qin et al. reported that lower circulating endogenous MOTS-c levels were associated with impaired coronary endothelial function in patients [45]. Another study revealed the lower level of MOTS-c in coronary artery disease (CAD) patients and a strong correlation between MOTS-c levels and CAD [46]. These results suggested a common theme of MOTS-c deficiency in cardiovascular pathology. Other studies identified both peptides as exercise-sensitive myokines, with their levels increasing in both blood stream and muscle following physical activity [26,47,48].

Our study extends these observations to AF. We found that HN and MOTS-c are downregulated in AF atrial tissue, and their deficiency correlates with fibrotic burden—a hallmark of AF progression. This local depletion is consistent with the concept that loss of these mitochondrial peptides may contribute to structural remodeling. We also observed decreased plasma MOTS-c in AF patients, which inversely correlated with NT-proBNP, a marker of cardiac wall stress. This aligns with the pattern seen in other cardiovascular conditions, further supporting the notion that low circulating MOTS-c may reflect cardiac injury. In contrast, plasma HN showed a slight elevation in our AF cohort, mirroring the discordant pattern seen in CKD [44]. This apparent paradox may reflect a compensatory systemic hormonal response to atrial stress, but direct evidence is lacking. This pattern suggests that plasma HN is not a reliable surrogate for atrial HN content and should not be interpreted as a simple biomarker for atrial pathology. We propose that plasma HN may be influenced by systemic factors such as inflammation, age, or other organ contributions.

Large amount of evidence confirmed that mitochondrial dysfunction, oxidative stress, and inflammation contribute to AF progression. Previous studies confirmed the cardioprotective potential of mitochondrial-derived peptides including HN and MOTS-c in some pathologic conditions. For instance, HN was shown to mitigate endothelial apoptosis [22] and age-related fibrosis [23], while MOTS-c can ameliorate pressure-overload heart failure [25] and diabetes-associated myocardial fibrosis [49]. The present study demonstrated that administration of both HN and MOTS-c can reduce AF inducibility and attenuate atrial fibrosis and hypertrophy induced by Ang II, suggesting their cardiac protective role in AF. Moreover, treatment with these peptides mitigate mitochondrial dysfunction, oxidative stress, and inflammation. Both peptides preserve mitochondrial ultrastructure and normalize the expression of fission proteins Drp1 and Fis1, which were reported to play important roles in stabilizing mitochondrial dynamics [50,51]. Notably, the upregulation of Drp1 aligns with recent single-cell RNA sequencing data from human AF atrial tissues, which revealed increased Drp1 expression across diverse cellular subpopulations [52]. By stabilizing mitochondrial dynamics, HNG and MOTS-c disrupt the consequent vicious cycle of oxidative stress and inflammation—key drivers of AF progression. The attenuation of this cycle is evidenced by reduced ROS, increased SOD activity, and downregulated IL-1β and IL-6, which is consistent with the anti-inflammatory effects of HNG reported in cerebral ischemia models [24]. Therefore, the improved mitochondrial function, downregulated oxidative stress, and reduced inflammation may attribute to the efficacy of administration of HN and MOTS-c in murine AF models.

Fibroblast activation plays a central role in the development of atrial fibrosis during atrial fibrillation [9,53,54]. The present data firstly revealed that both peptides directly inhibited AngII-induced transformation of fibroblasts into activated myofibroblasts, as well as their proliferation and migration. In murine models, administration of HNG and MOTS-c significantly attenuate fibrosis, suggesting their direct role on fibrosis. On the other hand, HNG and MOTS-c may also attenuate fibrosis in part by alleviating oxidative stress and inflammation, as well as improving the cardiomyocyte environment.

## 5. Limitations of the Study

Several limitations should be acknowledged. First, the human sample sizes are modest (39 matched pairs for plasma, 13 for atrial tissue), and the spatial transcriptomics dataset included only two donors per group. Larger, independent cohorts are needed to confirm our findings. Second, the AngII-infusion mouse model represents a subacute, uniform trigger and does not fully recapitulate the chronic, heterogeneous nature of clinical AF. We acknowledge that AF inducibility assessed by transesophageal burst pacing reflects susceptibility to triggered AF rather than spontaneous or persistent AF; thus, this model is useful for studying the early structural substrate but not chronic self-sustaining AF. Accordingly, our anti-AF claims are limited to the context of inducible AF in this subacute AngII model. Third, we did not include control groups receiving peptides alone without AngII; therefore, baseline effects of the peptides cannot be excluded. Fourth, the specific receptor(s) and immediate signaling pathways through which HN and MOTS-c act in cardiac cells remain unknown. Fifth, although we performed exploratory RNA-seq and qPCR validation, the functional relevance of the identified pathways requires further mechanistic investigation. Finally, the clinical significance of plasma MOTS-c as a biomarker is preliminary and requires validation in prospective studies with ROC analysis.

## 6. Conclusions

In summary, this study identifies downregulation of HN and MOTS-c as a novel feature of human AF that correlates with fibrosis. In a mouse model, exogenous supplementation with HNG or MOTS-c reduces AF susceptibility, preserves mitochondrial integrity, suppresses oxidative stress and inflammation, and directly inhibits fibroblast activation. These findings position the mitochondrial peptide pathway as a promising therapeutic target for AF, although further studies are required to establish causality and clinical utility.

## Data Availability

The RNA sequencing data of primary cardiac fibroblasts generated in this study have been deposited in the Gene Expression Omnibus (GEO) database under accession number GSE322635. The data are publicly available at the time of publication. The dataset GSE261363 is obtained from the GEO database (http://www.ncbi.nlm.nih.gov/geo/, accessed on 14 October 2024). Other data supporting the findings of this study are available from the corresponding author upon reasonable request.

## Supplementary Materials

The following supporting information can be downloaded at: https://www.mdpi.com/article/10.3390/biomedicines14051048/s1, Table S1. Baseline characteristics of the study population for plasma samples; Table S2. Baseline characteristics of the patients for atrial biopsies; Table S3. Primer sequences used in this study; Figure S1. Validation of RNA-seq-derived differentially expressed genes by qPCR in primary cardiac fibroblasts.

## Abbreviations

The following abbreviations are used in this manuscript:

AF	Atrial fibrillation
α-SMA	Alpha-smooth muscle actin
AngII	Angiotensin II
ANOVA	Analysis of variance
CAD	Coronary artery disease
CCK-8	Cell Counting Kit-8
Col1a1	Collagen type I alpha 1 chain
DAPI	4′,6-diamidino-2-phenylindole
DEGs	Differentially expressed genes
DHE	Dihydroethidium
DMEM	Dulbecco’s Modified Eagle Medium
DNA	Deoxyribonucleic acid
Drp1	Dynamin-related protein 1
ECG	Electrocardiogram
ELISA	Enzyme-linked immunosorbent assay
EPL	Eplerenone
FBS	Fetal bovine serum
Fis1	Mitochondrial fission protein 1
GEO	Gene Expression Omnibus
GO	Gene Ontology
HNG	Gly14-Humanin (Humanin analogue)
HN	Humanin
IF	Immunofluorescence
IHC	Immunohistochemistry
IL-1β	Interleukin-1 beta
IL-6	Interleukin-6
MDPs	Mitochondrial-derived peptides
MI	Myocardial infarction
MOTS-c	Mitochondrial open reading frame of the 12S rRNA-c
MRB	Mineralocorticoid receptor blocker
mRNA	Messenger RNA
MTRNR2L	MT-RNR2-like (nuclear humanin genes)
NRAMs	Neonatal rat atrial myocytes
NT-proBNP	N-terminal pro-brain natriuretic peptide
ox-LDL	Oxidized low-density lipoprotein
PAGE	Polyacrylamide gel electrophoresis
PBS	Phosphate-buffered saline
PCA	Principal component analysis
PCR	Polymerase chain reaction
PVDF	Polyvinylidene difluoride
qPCR	Quantitative polymerase chain reaction
RAAS	Renin–angiotensin–aldosterone system
RIPA	Radioimmunoprecipitation assay
RNA	Ribonucleic acid
ROS	Reactive oxygen species
SD	Standard deviation
SDS	Sodium dodecyl sulfate
SOD	Superoxide dismutase
SR	Sinus rhythm
TAC	Transverse aortic constriction
WGA	Wheat germ agglutinin

## References

[1] Marek-Iannucci S., Ozdemir A.B., Moreira D., Gomez A.C., Lane M., Porritt R.A., Lee Y., Shimada K., Abe M., Stotland A. (2021). Autophagy-mitophagy induction attenuates cardiovascular inflammation in a murine model of Kawasaki disease vasculitis. JCI Insight.

[2] Andrade J., Khairy P., Dobrev D., Nattel S. (2014). The clinical profile and pathophysiology of atrial fibrillation: Relationships among clinical features, epidemiology, and mechanisms. Circ. Res..

[3] Dzeshka M.S., Lip G.Y., Snezhitskiy V., Shantsila E. (2015). Cardiac Fibrosis in Patients with Atrial Fibrillation: Mechanisms and Clinical Implications. J. Am. Coll. Cardiol..

[4] Van Gelder I.C., Rienstra M., Bunting K.V., Casado-Arroyo R., Caso V., Crijns H., De Potter T.J.R., Dwight J., Guasti L., Hanke T. (2024). 2024 ESC Guidelines for the management of atrial fibrillation developed in collaboration with the European Association for Cardio-Thoracic Surgery (EACTS). Eur. Heart J..

[5] de Groot N.M., Houben R.P., Smeets J.L., Boersma E., Schotten U., Schalij M.J., Crijns H., Allessie M.A. (2010). Electropathological substrate of longstanding persistent atrial fibrillation in patients with structural heart disease: Epicardial breakthrough. Circulation.

[6] Krishnan A., Chilton E., Raman J., Saxena P., McFarlane C., Trollope A.F., Kinobe R., Chilton L. (2021). Are Interactions Between Epicardial Adipose Tissue, Cardiac Fibroblasts and Cardiac Myocytes Instrumental in Atrial Fibrosis and Atrial Fibrillation?. Cells.

[7] Zhu P., Li H., Zhang A., Li Z., Zhang Y., Ren M., Zhang Y., Hou Y. (2022). MicroRNAs sequencing of plasma exosomes derived from patients with atrial fibrillation: miR-124-3p promotes cardiac fibroblast activation and proliferation by regulating AXIN1. J. Physiol. Biochem..

[8] Karakasis P., Theofilis P., Vlachakis P.K., Korantzopoulos P., Patoulias D., Antoniadis A.P., Fragakis N. (2024). Atrial Fibrosis in Atrial Fibrillation: Mechanistic Insights, Diagnostic Challenges, and Emerging Therapeutic Targets. Int. J. Mol. Sci..

[9] Chen C., Chen P., Yu W., Zhao L., Yang Y., Qu H., Fu C., Shi D., Guo M. (2025). TGF-β-Driven Atrial Fibrosis in Atrial Fibrillation: From Mechanistic Insights to Targeted Therapies. Aging Dis..

[10] Anné W., Willems R., Holemans P., Beckers F., Roskams T., Lenaerts I., Ector H., Heidbüchel H. (2007). Self-terminating AF depends on electrical remodeling while persistent AF depends on additional structural changes in a rapid atrially paced sheep model. J. Mol. Cell. Cardiol..

[11] Ehrlich J.R., Hohnloser S.H., Nattel S. (2006). Role of angiotensin system and effects of its inhibition in atrial fibrillation: Clinical and experimental evidence. Eur. Heart J..

[12] Swedberg K., Zannad F., McMurray J.J., Krum H., van Veldhuisen D.J., Shi H., Vincent J., Pitt B. (2012). Eplerenone and atrial fibrillation in mild systolic heart failure: Results from the EMPHASIS-HF (Eplerenone in Mild Patients Hospitalization and SurvIval Study in Heart Failure) study. J. Am. Coll. Cardiol..

[13] Takemoto Y., Ramirez R.J., Kaur K., Salvador-Montañés O., Ponce-Balbuena D., Ramos-Mondragón R., Ennis S.R., Guerrero-Serna G., Berenfeld O., Jalife J. (2017). Eplerenone Reduces Atrial Fibrillation Burden Without Preventing Atrial Electrical Remodeling. J. Am. Coll. Cardiol..

[14] Murphy E., Ardehali H., Balaban R.S., DiLisa F., Dorn G.W., Kitsis R.N., Otsu K., Ping P., Rizzuto R., Sack M.N. (2016). Mitochondrial Function, Biology, and Role in Disease: A Scientific Statement from the American Heart Association. Circ. Res..

[15] Bukowska A., Schild L., Keilhoff G., Hirte D., Neumann M., Gardemann A., Neumann K.H., Röhl F.W., Huth C., Goette A. (2008). Mitochondrial dysfunction and redox signaling in atrial tachyarrhythmia. Exp. Biol. Med..

[16] Lin P.H., Lee S.H., Su C.P., Wei Y.H. (2003). Oxidative damage to mitochondrial DNA in atrial muscle of patients with atrial fibrillation. Free Radic. Biol. Med..

[17] Menezes Júnior A.D.S., França E.S.A.L.G., Oliveira J.M., Silva D.M.D. (2023). Developing Pharmacological Therapies for Atrial Fibrillation Targeting Mitochondrial Dysfunction and Oxidative Stress: A Scoping Review. Int. J. Mol. Sci..

[18] Xie W., Santulli G., Reiken S.R., Yuan Q., Osborne B.W., Chen B.X., Marks A.R. (2015). Mitochondrial oxidative stress promotes atrial fibrillation. Sci. Rep..

[19] Hashimoto Y., Niikura T., Tajima H., Yasukawa T., Sudo H., Ito Y., Kita Y., Kawasumi M., Kouyama K., Doyu M. (2001). A rescue factor abolishing neuronal cell death by a wide spectrum of familial Alzheimer’s disease genes and Abeta. Proc. Natl. Acad. Sci. USA.

[20] Lee C., Zeng J., Drew B.G., Sallam T., Martin-Montalvo A., Wan J., Kim S.J., Mehta H., Hevener A.L., de Cabo R. (2015). The mitochondrial-derived peptide MOTS-c promotes metabolic homeostasis and reduces obesity and insulin resistance. Cell Metab..

[21] Bodzioch M., Lapicka-Bodzioch K., Zapala B., Kamysz W., Kiec-Wilk B., Dembinska-Kiec A. (2009). Evidence for potential functionality of nuclearly-encoded humanin isoforms. Genomics.

[22] Bachar A.R., Scheffer L., Schroeder A.S., Nakamura H.K., Cobb L.J., Oh Y.K., Lerman L.O., Pagano R.E., Cohen P., Lerman A. (2010). Humanin is expressed in human vascular walls and has a cytoprotective effect against oxidized LDL-induced oxidative stress. Cardiovasc. Res..

[23] Qin Q., Mehta H., Yen K., Navarrete G., Brandhorst S., Wan J., Delrio S., Zhang X., Lerman L.O., Cohen P. (2018). Chronic treatment with the mitochondrial peptide humanin prevents age-related myocardial fibrosis in mice. Am. J. Physiol. Heart Circ. Physiol..

[24] Peng T., Wan W., Wang J., Liu Y., Fu Z., Ma X., Li J., Sun G., Ji Y., Lu J. (2018). The Neurovascular Protective Effect of S14G-Humanin in a Murine MCAO Model and Brain Endothelial Cells. IUBMB Life.

[25] Zhong P., Peng J., Hu Y., Zhang J., Shen C. (2022). Mitochondrial derived peptide MOTS-c prevents the development of heart failure under pressure overload conditions in mice. J. Cell. Mol. Med..

[26] Yuan J., Wang M., Pan Y., Liang M., Fu Y., Duan Y., Tang M., Laher I., Li S. (2021). The mitochondrial signaling peptide MOTS-c improves myocardial performance during exercise training in rats. Sci. Rep..

[27] Lu P., Li X., Li B., Li X., Wang C., Liu Z., Ji Y., Wang X., Wen Z., Fan J. (2023). The mitochondrial-derived peptide MOTS-c suppresses ferroptosis and alleviates acute lung injury induced by myocardial ischemia reperfusion via PPARγ signaling pathway. Eur. J. Pharmacol..

[28] Kavak A.G., Karslioglu I., Saracaloglu A., Demiryürek S., Demiryürek A.T. (2024). Impact of Radiation Therapy on Serum Humanin and MOTS-c Levels in Patients with Lung or Breast Cancer. Curr. Radiopharm..

[29] Li J., Wang S., Zhang Y.L., Bai J., Lin Q.Y., Liu R.S., Yu X.H., Li H.H. (2019). Immunoproteasome Subunit β5i Promotes Ang II (Angiotensin II)-Induced Atrial Fibrillation by Targeting ATRAP (Ang II Type I Receptor-Associated Protein) Degradation in Mice. Hypertension.

[30] Jiang J., Chang X., Nie Y., Xu L., Yang L., Peng Y., Chang M. (2023). Orally administered MOTS-c analogue ameliorates dextran sulfate sodium-induced colitis by inhibiting inflammation and apoptosis. Eur. J. Pharmacol..

[31] Gudiksen A., Hansen C.C., van der Stede T., Daugaard A.H., Schmidt J.H., Ringholm S., Merimi M., Al-Obaidi F.R., Kristoffersen A.T., Zole E. (2026). MOTS-c improves intrinsic muscle mitochondrial bioenergetic health and efficiency in a PGC-1α/AMPK-dependent manner. Free Radic. Biol. Med..

[32] Li D.S., Xue G.L., Yang J.M., Li C.Z., Zhang R.X., Tian T., Li Z., Shen K.W., Guo Y., Liu X.N. (2022). Knockout of interleukin-17A diminishes ventricular arrhythmia susceptibility in diabetic mice via inhibiting NF-κB-mediated electrical remodeling. Acta Pharmacol. Sin..

[33] Han W., Du C., Zhu Y., Ran L., Wang Y., Xiong J., Wu Y., Lan Q., Wang Y., Wang L. (2022). Targeting Myocardial Mitochondria-STING-Polyamine Axis Prevents Cardiac Hypertrophy in Chronic Kidney Disease. JACC Basic. Transl. Sci..

[34] Schrickel J.W., Bielik H., Yang A., Schimpf R., Shlevkov N., Burkhardt D., Meyer R., Grohé C., Fink K., Tiemann K. (2002). Induction of atrial fibrillation in mice by rapid transesophageal atrial pacing. Basic Res. Cardiol..

[35] Murphy M.B., Kim K., Kannankeril P.J., Subati T., Van Amburg J.C., Barnett J.V., Murray K.T. (2022). Optimizing transesophageal atrial pacing in mice to detect atrial fibrillation. Am. J. Physiol. Heart Circ. Physiol..

[36] Davis S., Meltzer P.S. (2007). GEOquery: A bridge between the Gene Expression Omnibus (GEO) and BioConductor. Bioinformatics.

[37] Ritchie M.E., Phipson B., Wu D., Hu Y., Law C.W., Shi W., Smyth G.K. (2015). Limma powers differential expression analyses for RNA-sequencing and microarray studies. Nucleic Acids Res..

[38] Hao D., Yang X., Li Z., Xie B., Feng Y., Liu G., Ren X. (2025). Screening core genes for minimal change disease based on bioinformatics and machine learning approaches. Int. Urol. Nephrol..

[39] Chin Y.P., Keni J., Wan J., Mehta H., Anene F., Jia Y., Lue Y.H., Swerdloff R., Cobb L.J., Wang C. (2013). Pharmacokinetics and tissue distribution of humanin and its analogues in male rodents. Endocrinology.

[40] Wang W., Li E., Zou J., Qu C., Ayala J., Wen Y., Islam M.S., Weintraub N.L., Fulton D.J., Liang Q. (2024). The Ubiquitin Ligase RBX2/SAG Regulates Mitochondrial Ubiquitination and Mitophagy. Circ. Res..

[41] Zahid M.A., Abdelsalam S.S., Raïq H., Parray A., Korashy H.M., Zeidan A., Elrayess M.A., Agouni A. (2023). Sestrin2 as a Protective Shield against Cardiovascular Disease. Int. J. Mol. Sci..

[42] Cai H., Cao P., Sun W., Shao W., Li R., Wang L., Zou L., Forno E., Muzumdar R., Gong Z. (2022). Circulating humanin is lower in coronary artery disease and is a prognostic biomarker for major cardiac events in humans. Biochim. Biophys. Acta Gen. Subj..

[43] Conte M., Ostan R., Fabbri C., Santoro A., Guidarelli G., Vitale G., Mari D., Sevini F., Capri M., Sandri M. (2019). Human Aging and Longevity Are Characterized by High Levels of Mitokines. J. Gerontol. A Biol. Sci. Med. Sci..

[44] Liu C., Gidlund E.K., Witasp A., Qureshi A.R., Söderberg M., Thorell A., Nader G.A., Barany P., Stenvinkel P., von Walden F. (2019). Reduced skeletal muscle expression of mitochondrial-derived peptides humanin and MOTS-C and Nrf2 in chronic kidney disease. Am. J. Physiol. Ren. Physiol..

[45] Qin Q., Delrio S., Wan J., Jay Widmer R., Cohen P., Lerman L.O., Lerman A. (2018). Downregulation of circulating MOTS-c levels in patients with coronary endothelial dysfunction. Int. J. Cardiol..

[46] Yaşar E., Çakmak T., Bayramoğlu A., Karakuş Y., Tekin S., Şekerci G., Türkoğlu C. (2022). MOTS-c as a predictor of coronary lesions and complexity in patients with stable coronary artery disease. Eur. Rev. Med. Pharmacol. Sci..

[47] Woodhead J.S.T., D’Souza R.F., Hedges C.P., Wan J., Berridge M.V., Cameron-Smith D., Cohen P., Hickey A.J.R., Mitchell C.J., Merry T.L. (2020). High-intensity interval exercise increases humanin, a mitochondrial encoded peptide, in the plasma and muscle of men. J. Appl. Physiol. (1985).

[48] Reynolds J.C., Lai R.W., Woodhead J.S.T., Joly J.H., Mitchell C.J., Cameron-Smith D., Lu R., Cohen P., Graham N.A., Benayoun B.A. (2021). MOTS-c is an exercise-induced mitochondrial-encoded regulator of age-dependent physical decline and muscle homeostasis. Nat. Commun..

[49] Li Z., Wang T., Fu Y., Chen F., Li S. (2026). Aerobic exercise and MOTS-c attenuate diabetic myocardial fibrosis via inhibition of the THBS1/TGF-β signaling pathway. Front. Endocrinol..

[50] Tilokani L., Nagashima S., Paupe V., Prudent J. (2018). Mitochondrial dynamics: Overview of molecular mechanisms. Essays Biochem..

[51] Quiles J.M., Gustafsson Å.B. (2022). The role of mitochondrial fission in cardiovascular health and disease. Nat. Rev. Cardiol..

[52] Li Y., Liu X., Lin R., Peng X., Wang X., Meng F., Jin S., Lv W., Liu X., Du Z. (2024). Ibrutinib Promotes Atrial Fibrillation by Disrupting A-Kinase Anchoring Protein 1-Mediated Mitochondrial Quality Surveillance in Cardiomyocytes. Research.

[53] Harada M., Nattel S. (2021). Implications of Inflammation and Fibrosis in Atrial Fibrillation Pathophysiology. Card. Electrophysiol. Clin..

[54] Schotten U., Goette A., Verheule S. (2025). Translation of pathophysiological mechanisms of atrial fibrosis into new diagnostic and therapeutic approaches. Nat. Rev. Cardiol..

