# Supplementary material for: Humanin and MOTS-c Attenuate Atrial Fibrillation by Suppressing Fibrosis and Mitochondrial Dysfunction
Source: Biomedicines. 2026 May 5;14(5):1048. doi: 10.3390/biomedicines14051048 (PMC13204372; doi:10.3390/biomedicines14051048)
Supplement: Supplementary file 1 [file biomedicines-14-01048-s001.zip › biomedicines-4241763-supplementary.pdf]

Supplemental materials

Humanin and MOTS-c Attenuate Atrial Fibrillation by Suppressing Fibrosis and Mitochondrial Dysfunction

Supplemental Tables

Table S1 Baseline characteristics of the study population for plasma samples.

	SR (n=39)	AF (n=39)	P value
Age (years) (Mean ± SD)	74.15 ± 6.36	73.41 ± 5.45	0.581
Male gender, n (%)	24 (61.5)	21 (53.8)	0.647
EF (%) (Mean ± SD)	59.28 ± 3.68	57.97 ± 5.62	0.237
Diabetes, n (%)	11 (28.2)	14 (35.9)	0.811
Hypertension, n (%)	27 (69.2)	27 (69.2)	1.000
NT-proBNP (pg/ml) (Mean ± SD)	118.69 ± 92.71	969.41 ± 580.25	<0.001
Humanin (pg/ml) (Mean ± SD)	60.37 ± 31.78	77.11 ± 37.92	0.038
MOTS-c (pg/ml) (Mean ± SD)	188.85 ± 125.69	93.72 ± 38.65	<0.001
BMI ($kg·m^{-2}$) (Mean ± SD)	25.21 ± 3.60	25.66 ± 2.91	0.639
Waist hip rate (Mean ± SD)	0.91 ± 0.08	0.92 ± 0.06	0.876
Smoking history, n (%)	9 (23.1)	3 (7.7)	0.117
Drinking history, n (%)	4 (10.3)	3 (7.7)	1.000
Scr (umol/L) (Mean ± SD)	73.89 ± 18.01	73.84 ± 19.66	0.990
Medications			
RAAS inhibitors, n (%)	15 (38.5)	15 (38.5)	1.000
β-blockers, n (%)	2 (5.1)	16 (41.0)	<0.001
Statins, n (%)	4 (10.3)	8 (20.5)	0.347
Anticoagulants, n (%)	0 (0.00)	16 (41.03)	<0.001

SR, sinus rate; AF, atrial fibrillation; EF, ejection fraction; BMI, body mass index; Scr, serum creatinine; RAAS, Renin-angiotensin-aldosterone system.

Table S2 Baseline characteristics of the patients for atrial biopsies.

	SR (n=6)	AF (n=7)	P value
Age (years) (Mean ± SD)	60.00 ± 16.12	68.00 ± 7.67	0.306
Male gender, n (%)	5 (83.33)	4 (57.14)	0.559
**The type of AF**			
Persistent atrial fibrillation	0/6	4/7	
Paroxysmal atrial fibrillation	0/6	3/7	

SR, sinus rate; AF, atrial fibrillation.

Table S3 Primer sequences used in this study.

Species	Target genes	Forward primers (5'-3')	Reverse primers (5'-3')
Rat	α-SMA	CCACTGCTGCTTCCTCTTCTTC	TGCCCGCCGACTCCATTC
	Col1a1	TGTTGGTCCTGCTGGCAAGAATG	GTCACCTTGTTCGCCTGTCTCAC
	β-Actin	TGCTATGTTGCCCTAGACTTCG	GTTGGCATAGAGGTCTTTACGG
	Prelp	ATTCGCAAGGTGGACCAGAG	ATCTGGGTCCCATTGATCTTCTC
	Sox10	CACGCAGAAAGTTAGCCGAC	TCTCGTTCAGCAACCTCCAGA
	Gpld1	TTCGGCTGGTGATTTTGGAGG	CTAGCATCTCCCCGTGCATT
	Map3k15	GCGAAAGTTCGCAGGGGG	GTCCACAATGGCAACATCTGC
	Ackr4	AGTCTTCCTGCCTGCCTTCTTC	ACACATCGGTCTTGGTCCTCTG
	Cx3cr1	CGCAACTCGGAGGTCAACATC	AAGACAACAACCACCAAGAGGATG
	Hspa1l	ACCGTGCCAGCCTATTTCAATG	TGCCGCTCTCCGTGACTTC
	Nudt11	AGCAGAACCAGGACCGCAAG	GGCATCTTCGATCTTGAACCACTC
Mouse	Nppa	GCTTCCAGGCCATATTGGAG	GGGGGCATGACCTCATCTT
	Nppb	GAGGTCACTCCTATCCTCTGG	GCCATTTCCTCCGACTTTTCTC
	Acta1	AATGAGCGTTTCCGTTGC	ATCCCCGCAGACTCCATAC
	Myh7	CGCATCAAGGAGCTCACC	CTGCAGCCGCAGTAGGTT
	Sesn2	GAGTGCCATTCCGAGATCAAG	TAGTCCGGGTGTAGACCCATC
	Drp1	TAAGCCCTGAGCCAATCCATC	CATTCCCGGTAAATCCACAAGT
	Fis1	AGGCTCTAAAGTATGTGCGAGG	GGCCTTATCAATCAGGCGTTC
	IL-1β	ACCTGCTGGTGTGTGACGTT	TCGTTGCTTGGTTCTCCTTG
	IL-6	GCCTTCTTGGGACTGATGCT	TCTGTGACTCCAGCTTATCTCTTG
	β-Actin	ACCTTCTACAATGAGCTGCG	CTGGATGGCTACGTACATGG

Supplemental Figure

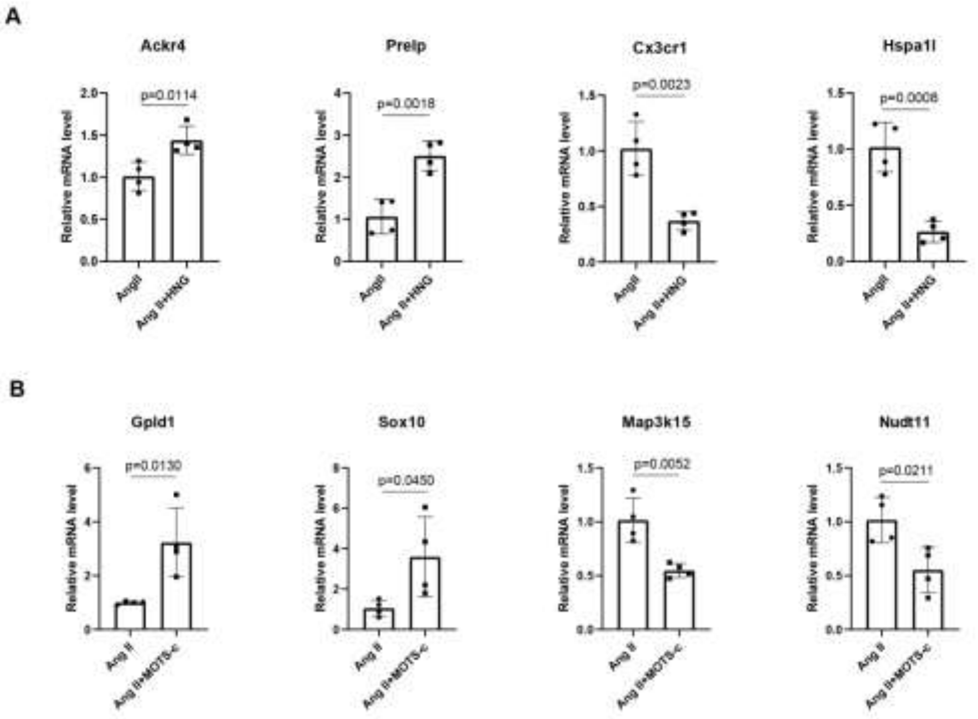

Figure s1. Validation of RNA-seq-derived differentially expressed genes by qPCR in primary cardiac fibroblasts. (A) qPCR validation of two upregulated genes (Ackr4, Prelp) and two downregulated genes (Cx3cr1, Hspa1l) identified from the AngII vs. AngII+HNG comparison. (B) qPCR validation of two upregulated genes (Gpld1, Sox10) and two downregulated genes (Map3k15, Nudt11) identified from the AngII vs. AngII+MOTS-c comparison. Data in A, B were analyzed via the Student t tests. n = 4 independent biological replicates per group. The qPCR results confirm the direction of change for the selected DEGs, supporting the reliability of the RNA-seq findings.
